# Targeted Drug Screening Leveraging Senescence-Induced T-Cell Exhaustion Signatures in Hepatocellular Carcinoma

**DOI:** 10.3390/ijms252011232

**Published:** 2024-10-18

**Authors:** Qi Qi, Jianyu Pang, Yongzhi Chen, Yuheng Tang, Hui Wang, Samina Gul, Yingjie Sun, Wenru Tang, Miaomiao Sheng

**Affiliations:** Laboratory of Molecular Genetics of Aging & Tumor, Medicine School, Kunming University of Science and Technology, Kunming 650500, China; qiqi12047@163.com (Q.Q.); jianyu_0898@163.com (J.P.); cyz1206414925@163.com (Y.C.); t1023063288@163.com (Y.T.); huiwang266@163.com (H.W.); saminagul@kust.edu.cn (S.G.); sunyj6039@163.com (Y.S.)

**Keywords:** hepatocellular carcinoma, cellular senescence, t cell exhaustion, machine learning, single-cell RNA-seq, combined targeted therapy

## Abstract

Hepatocellular carcinoma (HCC) is the sixth most prevalent cancer and a leading cause of cancer-related mortality globally, with most patients diagnosed at advanced stages and facing limited early treatment options. This study aimed to identify characteristic genes associated with T-cell exhaustion due to senescence in hepatocellular carcinoma patients, elucidating the interplay between senescence and T-cell exhaustion. We constructed prognostic models based on five signature genes (ENO1, STMN1, PRDX1, RAN, and RANBP1) linked to T-cell exhaustion, utilizing elastic net regression. The findings indicate that increased expression of ENO1 in T cells may contribute to T-cell exhaustion and Treg infiltration in hepatocellular carcinoma. Furthermore, molecular docking was employed to screen small molecule compounds that target the anti-tumor effects of these exhaustion-related genes. This study provides crucial insights into the diagnosis and treatment of hepatocellular carcinoma, establishing a strong foundation for the development of predictive biomarkers and therapeutic targets for affected patients.

## 1. Introduction

Hepatocellular carcinoma (HCC) is the sixth most common cancer in the world, accounting for more than 80% of primary liver cancer cases globally and the third leading cause of cancer-related deaths [1]. It is generally categorized into primary hepatocellular carcinoma and secondary hepatocellular carcinoma [2]. The two main types of primary liver cancer are hepatocellular carcinoma and intrahepatic cholangiocarcinoma (ICC), and less commonly angiosarcoma, hemangiosarcoma, and hepatoblastoma. ICC originates in the bile ducts, whereas HCC originates in the hepatocytes, which are the main parenchymal cells of the liver [3]. Currently, the primary approaches in clinical practice for treating HCC include hepatic artery chemoembolization, hepatic resection, percutaneous thermal ablation, radiotherapy, systemic therapy, and immunotherapy [4,5]. Recent studies have demonstrated that immune checkpoint molecules play a crucial role in tumor immune escape, and the investigation of their blocking mechanisms has yielded promising therapeutic results, gradually transforming traditional cancer treatment approaches [6]. This underscores the need for the development of additional biomarkers for clinical guidance [7].

The tumor microenvironment of HCC is a complex mixture characterized by aberrant angiogenesis, chronic inflammation, and dysregulated extracellular matrix remodeling, creating an immunosuppressive environment that promotes HCC proliferation, invasion, and metastasis [8]. The heterogeneity of immune cells and the complexity of the tumor microenvironment are key factors influencing the response and prognosis of HCC. It is well known that a class of T cells called cytotoxic T cells, also known as CD8+ T cells, can directly kill infected or mutated cells. However, T cell dysfunction in tumors is usually associated with the accumulation of T-cell exhaustion (Tex cells) that remain alive but with reduced effector activity [9]. Moreover, the assessment of T-cell exhaustion has different important roles at different times. It has been suggested that evidence of CD8+ T-cell exhaustion in pre-treatment and early treatment tumor samples can predict the clinically benign effect of Immune checkpoint inhibitor therapy [10].

Cellular senescence is a stable, growth-arrested state that results from endogenous or exogenous stimuli, including telomere dysfunction, oncogene activation, and persistent DNA damage [11]. During senescence, the immune system loses the ability to respond effectively to pathogens and cancer cells, a decline in immune function known as immunosenescence [12]. Aging leads to T cell immune remodeling, which can trigger cancer and age-related diseases, and is generally associated with poor clinical outcomes. Therefore, it is crucial to investigate the relationship between senescence-induced T-cell exhaustion and antitumor immunity [13]. The cellular senescence markers studied, once validated across diverse populations and environments, will serve as powerful tools for monitoring healthy aging, screening for age-related diseases, and identifying longevity interventions [14].

In recent years, small molecule drugs targeting prognostic genes have garnered significant attention in cancer therapy. These compounds can regulate tumor growth and metastasis by specifically targeting key genes and signaling pathways. Osteotriol (Vitamin D3), Astragaloside C, Gentamicin, and Ivermectin are notable small molecule agents that have been investigated for their potential anti-tumor effects. Osteotriol, the active form of vitamin D, has demonstrated inhibitory effects across various cancer types [15]. Astragaloside C, a component of traditional Chinese medicine, has shown promising immunomodulatory and anti-tumor properties [16]. While Gentamicin is primarily recognized for its anti-infective applications, emerging studies indicate its capacity to inhibit tumor growth in specific cancer models [17]. Additionally, Ivermectin, initially developed as an antiparasitic agent, has been widely reported to possess the ability to inhibit viral replication in vitro [18]. Several studies suggest that Ivermectin may serve as a potentially effective drug for combating viral infections, including COVID-19 [19,20].

This study focuses on immunotherapy, exploring the tumor immune microenvironment of T-cell exhaustion in hepatocellular carcinoma using single-cell data. It dissects the effects of senescence on T-cell exhaustion, identifies potential prognostic genes associated with senescence-induced T-cell exhaustion, and seeks drug targets for combination therapy to enhance clinical outcomes in hepatocellular carcinoma.

## 2. Results

### 2.1. Construction of Human Hepatocellular Carcinoma T-Cell Profiles at Single-Cell Resolution

**Figure 1:**
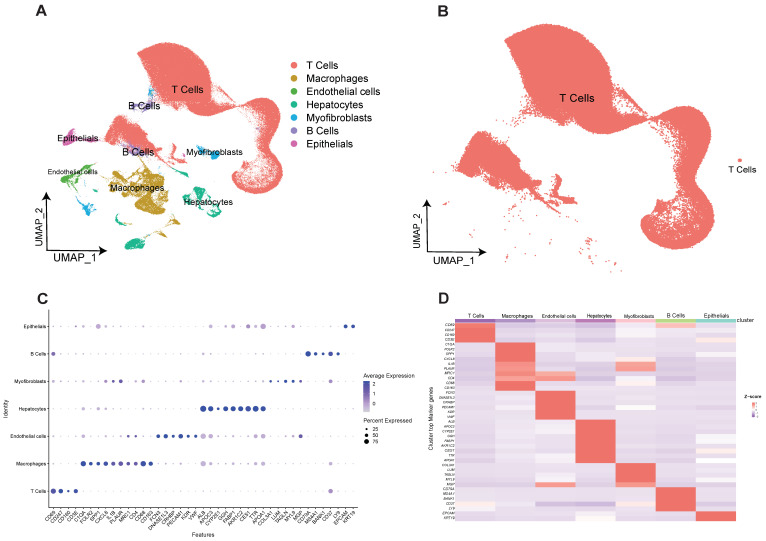
Construction of human hepatocellular carcinoma T-cell profiles at single-cell resolution. (**A**) Obtained subgroup clustering subgroups for seven cell types. (**B**) 170,000 T cells clustered together due to transcriptional similarity. (**C**) Seven cell clusters with expression levels of marker genes are shown. (**D**) Heatmap of the expression level of Marker genes from seven cell types.

The four scRNA-seq datasets included in this study were GSE162616, GSE166635, GSE175783, and GSE149614. Low-quality cells were removed and integrated using anchors, and a total of 229,458 cells were included in the study. After UMAP and TSNE downscaling, 28 cell clusters were obtained. Next, we analyzed the known marker genes for the major expected cell types by the characteristic genes of the cells as well as the reports in the available literature, and further obtained subgroup clustering subgroups for seven cell types, namely T cells, macrophages, B cells, hepatocytes, epithelial cells/cancer cells, endothelial cells, and myofibroblasts (Figure 1A). Among them, B cells and T cells were partially clustered due to transcriptional similarity. Meanwhile, because of the heterogeneity of T-cell phenotype and function, the total number of extracted cells was about 170,000 and the distribution was not significantly aggregated (Figure 1B). Seven major cell clusters with expression levels of marker genes are shown in figure (Figure 1C), indicating that different cell types have their own specific marker genes (Figure 1D). Cell annotation information is shown in Supplementary Materials Table S1 Cell cluster annotation information.

### 2.2. Differential Characterization of Aging and Young T Cell Subsets

**Figure 2:**
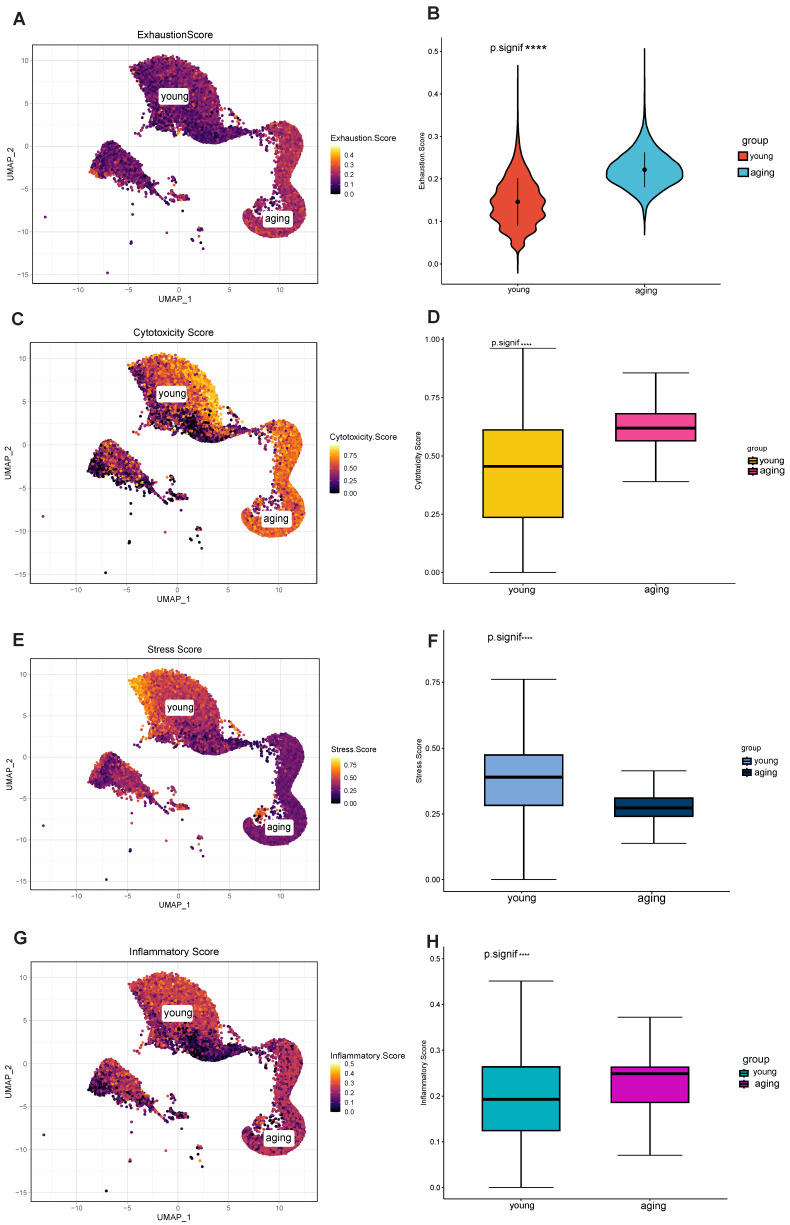
Differential characterization of senescent and young T cell subsets. (**A**) Senescent and young T cell populations, and calculated exhaustion scores. (**B**) Significantly different. (**C**) The cytotoxicity score of the senescent group was significantly higher than that of the young group. (**D**) Significantly different. (**E**) The stressful score of the senescent group was significantly higher than that of the young group. (**F**) Significantly different. (**G**) The senescent T-cell subpopulation showed relatively high inflammation scores. (**H**) Significantly different. **** *p* < 0.0001.

Next, we further examined gene expression profiles to decipher the functional differences between different T cell populations. In this study, we utilized the senescence gene set from the Human Senescence Genome Database to classify T cells into young and senescent populations, investigating the relationship between senescent T cells and exhaustion markers. While there is a connection between the two, they represent distinct biological events. The ssGSEA algorithm was utilized to classify 170,000 T cells into senescent and young categories. Subsequently, the AUCell algorithm was employed to calculate the exhaustion scores (Figure 2A,B). Then, the different properties of aging and young T cell subpopulations were explored in terms of cytotoxicity, stressful pressure, and degree of inflammation.

In terms of cytotoxicity, the cytotoxicity score of the aging group was significantly higher than that of the young group (Figure 2C), and this difference was statistically significant (Figure 2D). Some forms of cytotoxicity may accelerate cellular senescence or lead to cellular exhaustion, while cellular senescence and exhaustion may also increase sensitivity to toxic factors [21]. Significantly, in terms of stressful pressures, the younger group exhibited higher stress scores (Figure 2E,F). This suggests that the younger group was more adaptive and resilient in response to stress and challenges compared to the senescent group, prompting the cells to undergo the aging process faster. As for the degree of inflammation, the senescent T-cell subpopulation showed relatively high inflammation scores (Figure 2G,H) and predominantly expressed CCL3, CCL4, and CCL4L2, suggesting its ability to recruit other immune cells such as T cells [22]. Inflammatory cytokines enhance immunity by guiding naïve CD8+ T cells to sites of interaction with CD4+ T cells and dendritic cells. Yet, the frequency of both antigen-bearing cells and relevant naïve T cells is low during early infection. This suggests that active mechanisms promote the necessary cellular interactions.

Taken together, we analyzed the distinct properties of aging and young T cell subsets. In contrast to young T cells, senescent T cells exhibited higher cytotoxicity and lower stress scores, indicating significantly different transcriptional and functional phenotypes.

### 2.3. Acquisition of a Set of Genes Highly Associated with Aging T-Cells

**Figure 3:**
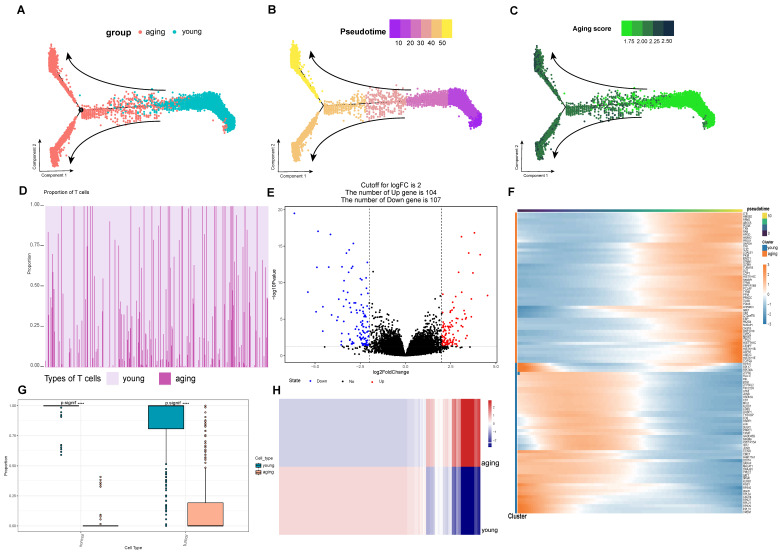
Screening of gene sets highly associated with senescent T cells. (**A**) The senescent and young T cell differentiation trajectories. (**B**) The calculation was consistent with the monocle algorithm. (**C**) Verified the pseudotime axis of ssGSEA on the senescence scoring of a single sample. (**D**) The abundance of senescent and young T cells in the samples using data from 424 samples from the TCGA-LIHC cohort. (**E**) With 104 genes up-regulated in the senescent group. (**F**) The TOP50 feature genes were expressed as shown. (**G**) Significant difference in the proportion of senescent and young T cells in the tumor group. (**H**) The DEG differential gene calculation resulted in a heat map of expression between the two groups. **** *p* < 0.0001.

Next, we used Monocle to analyze the pseudotime trajectories of T cell subpopulations. Based on the senescence genes given by the official website of Human Aging Genomic Resources (https://genomics.senescence.info/, accessed on 14 April 2023), we distinguished the aging and young T cell differentiation trajectories (Figure 3A), with young T cell populations at the beginning and aging T cell populations at the end, which is a significant effect of subpopulation. And the results of ssGSEA senescence scoring of individual samples were validated on the proposed time-series axis (Figure 3C), and the computational results were consistent with the monocle algorithm (Figure 3B). The FindAllMarkers function was used to calculate the feature genes in different trajectories, in which the TOP50 feature genes were expressed as shown (Figure 3F), such as the LTB gene (lymphotoxin β) which is a potential biomarker for lymphocyte differentiation [23]. Ultimately, we identified signature genes in the T-cell exhaustion trajectory in hepatocellular carcinoma, which we defined as the T-cell exhaustion-related genes (TERGs).

On the other hand, in this study we calculated the abundance of aging and young T cells in the samples using data from 424 samples from the TCGA-LIHC cohort (Figure 3D). At the same time, we found that there was a significant difference in the proportion of aging and young T cells in the tumor group (Figure 3G). The result of the DEG differential gene calculation resulted in a heat map of expression between the two groups (Figure 3H), and it was obvious that there was a significant differentiation in the expression levels in the two cellular taxa, with 104 genes up-regulated in the aging group (Figure 3E), and we defined the up-regulated differential genes as the differential gene set for T-cell exhaustion (DEGs).

### 2.4. Establishment of a Stable and Effective Model of T-Cell Prognostic Exhaustion

The TCGA database included a total of 424 samples, with 368 samples in the tumor cohort (n = 368). The dataset was randomly split in a 2:1 ratio, resulting in a training set (n = 245) and an internal validation set (n = 175). Prognostic risk models were constructed using concatenated genes from tumor endothelial-related genes (TERGs) and differentially expressed genes (DEGs). This concatenated gene risk model highlighted significant roles in T-cell exhaustion and senescence. Five key prognostic genes were identified, and their coefficients were determined using the Elastic Net Regression algorithm. The prognostic exhaustion model (TERGs) was defined as:

TERG score = 0.014 × ENO1_Exp_ + 0.016 × STMN1_Exp_ + 0.038 × PRDX1_Exp_ + 0.06 × RAN_Exp_ + 0.06 × RANBP1_Exp_.

**Figure 4:**
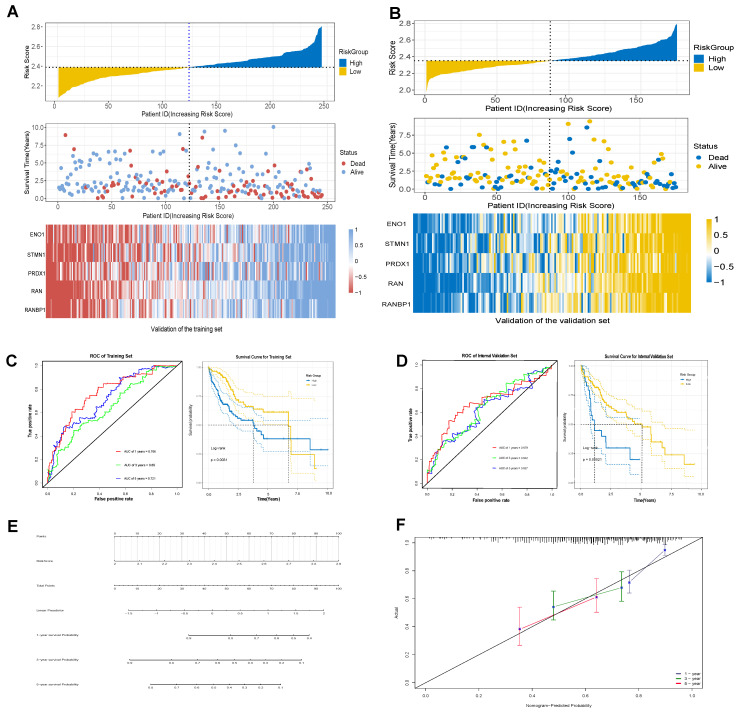
Establishment of model of T-cell prognostic exhaustion. (**A**) Gene expression in the training set. (**B**) Gene expression in the validation set. (**C**) The training set of ROC and Kaplan–Meier survival curves. (**D**) The validation set of ROC and Kaplan–Meier survival curves. (**E**) The nomogram of prognostic characteristics. (**F**) The nomogram calibration curves to predict the 1-, 3-, and 5-year survival rates.

Time-dependent ROC curves evaluated the independent prognostic ability of the exhaustion model. The AUC values for 1, 3, and 5 years in both the training and internal validation sets exceeded 0.6, indicating strong predictive capability for hepatocellular carcinoma survival (Figure 4C,D). Similarly, in both the training and validation sets, the heat maps clearly demonstrated significant differential expression of prognostic genes between high and low risk groups (Figure 4A,B). Kaplan–Meier survival curves showed statistically significant differences in survival between the cohorts based on exhaustion scores (Log-rank, *p* < 0.001), suggesting exhaustion scores as reliable prognostic indicators (Figure 4C,D).

Additionally, the nomogram of prognostic characteristics was examined (Figure 4E), with calibration curves for 1-year, 3-year, and 5-year survival rates, demonstrating a high degree of overlap between actual survival rates and those predicted by the nomogram, indicating exceptional predictive value (Figure 4F).

### 2.5. High Levels of TERGs Are Associated with a Tumor-Suppressive Microenvironment

**Figure 5:**
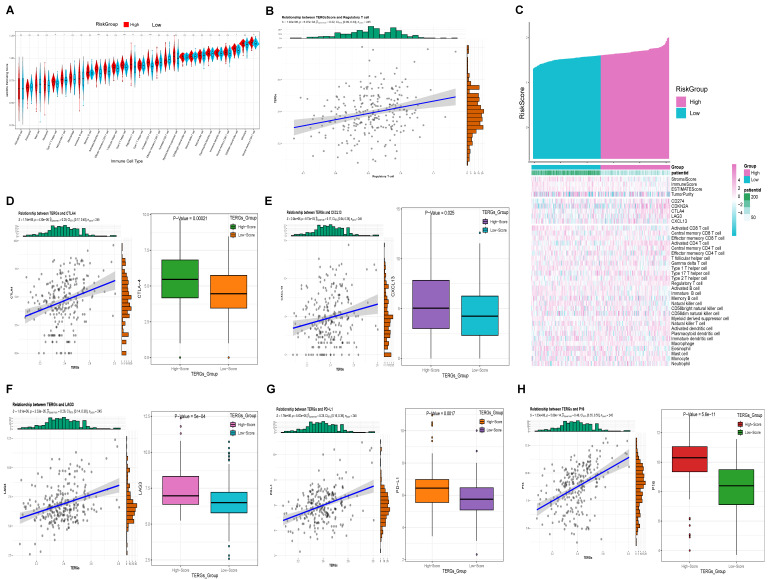
TERGs are associated with a tumor-suppressive microenvironment. (**A**) ssGSEA results showed that there were eight types of immune cells. (**B**) Tregs cell infiltration was increased. (**C**) The higher immune component of the tumor microenvironment in the group with high T-cell exhaustion. (**D**–**H**) Immune checkpoints (PD-L1, CTLA-4, LAG-3, CXCL13) and p16 with elevated expression in high TERGs.

We conducted a detailed investigation of the tumor immune microenvironment associated with TERGs. ssGSEA results indicated that eight types of immune cells were differentiated between the high and low groups of the TERGs: Type 17 T helper cells, natural killer T cells, activated dendritic cells, Type 2 T helper cells, regulatory T cells, activated CD4 T cells, CD56dim natural killer cells, and central memory CD4 T cells. Among these, activated dendritic cells, Type 2 T helper cells, and activated CD4 T cells were particularly significant, followed by regulatory T cells, Type 17 T helper cells, and central memory CD4 T cells (Figure 5A). We observed that the high TERGs group were significantly higher than those of the low group, likely due to the increased immune components in the tumor microenvironment of the high group. This finding suggests that the immune prognosis in the high group is more complex (Figure 5C). Notably, correlation analysis indicated that activated CD4 T cells, Type 17 T helper cells, Type 2 T helper cells, regulatory T cells, and activated dendritic cells all exhibited significant positive correlations with TERGs (Figure S1). Among these, regulatory T cells showed particularly strong significance. In Figure 5B, we observed a significant positive correlation between TERGs and Tregs, with an increased infiltration of Tregs in the High group. This suggests that TERGs contribute to a tumor-suppressive microenvironment.

Furthermore, we identified four classic immune checkpoints (CTLA-4, CXCL13, LAG-3, PD-L1) (Figure 5D–G) and the senescent marker P16 (Figure 5H) that exhibited elevated expression in the high TERGs group, aligning with our previous findings on immune infiltration. Prior studies have suggested a pro-tumorigenic role for CCL4+ and PD-L1 in immune cells, particularly tumor-associated neutrophils [24]. Exhausted T cells in the late stages of cellular differentiation are typically characterized by elevated expression of exhaustion-related genes, including PD-1, CTLA-4, LAG-3, TIM-3, and CD39, along with high levels of chemokines such as CXCL13 [25]. Therefore, our hypothesis is that the higher the TERGs, the deeper the senescence, the higher the T-cell exhaustion.

### 2.6. Clinical Applications of Exhaustion Models

**Figure 6:**
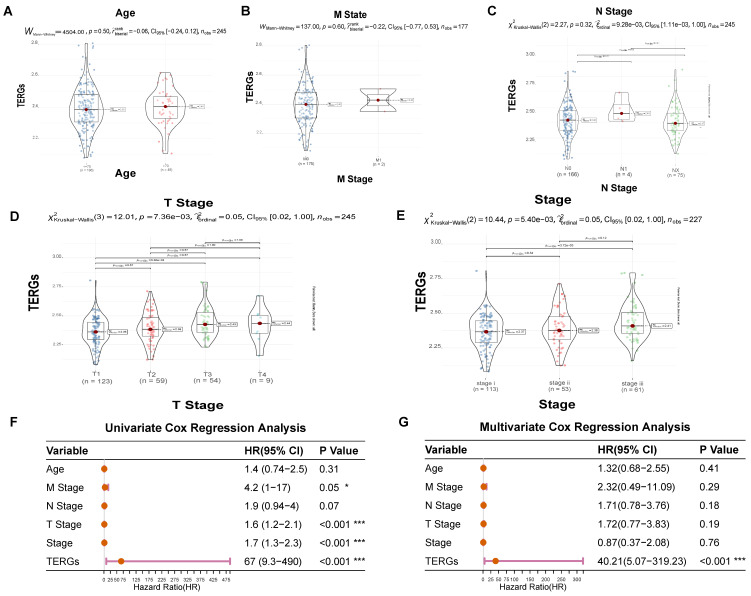
The relationship between clinical features and exhaustion score. (**A**–**E**) Age, M stage, N stage, T stage, and stage distribution of the patients in the high-risk and low-risk groups. (**F**) Univariate Cox Regression analysis and (**G**) Multivariate Cox Regression analysis of clinical information of TCGA cohorts (* *p* < 0.05; *** *p* < 0.001).

We further investigated the relationship between clinical features and exhaustion score (Figure 6A–E). Age, M stage, and N stage do not exhibit statistically significant differences in exhaustion scores, suggesting that there is no direct correlation between these factors and the degree of exhaustion (Figure 6A–C). However, within the TNM tumor staging system, significant statistical differences were observed among T stages, indicating a correlation between exhaustion scores and T stages (Figure 6D). The overall stage reflects the extent of lymph node involvement and metastasis. Notably, as TERGs increase, the severity of Stage I, Stage II, and Stage III also escalates (Figure 6E). Higher exhaustion scores are linked to larger primary tumors, increased invasion of surrounding tissues, greater overall damage, and a poorer prognosis.

Next, we employed univariate and multivariate Cox regression analyses to assess the clinical utility of exhaustion models in predicting patient prognosis. The univariate Cox analysis revealed that T stage, overall stage, and TERGs were significantly associated with prognosis, with TERGs demonstrating particularly strong significance (*p* < 0.001, HR = 67, 95% CI 9.3–490), indicating a poor prognosis (Figure 6F). Furthermore, the multivariate Cox results also affirmed that exhaustion score is an effective independent prognostic factor for LIHC (*p* < 0.001, HR = 40.21, 95% CI 5.07~319.23) (Figure 6G). These findings strongly support the robust and independent prognostic value of the TERGs index concerning T-cell exhaustion.

### 2.7. Probing the Function of Five Prognostic Genes Associated with Senescent T-Cells

**Figure 7:**
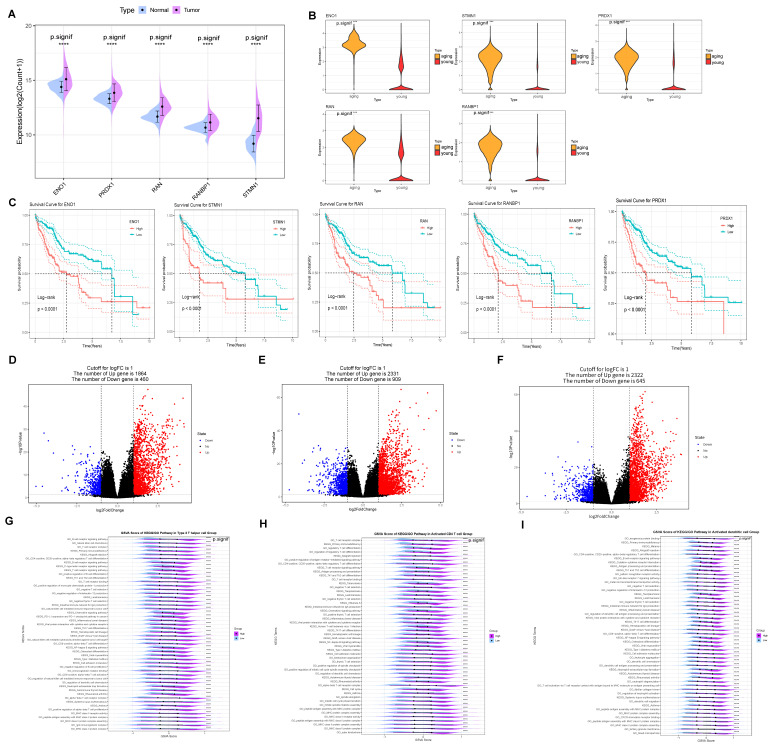
The function of five prognostic genes associated with senescent T cells. (**A**) ENO1 expression was up-regulated in the tumor group of TCGA cohorts and (**B**) significantly up-regulated in senescent T cells compared to the young group. (**C**) ENO1, STMN1, PRDX1, RANBP1, and RAN high expression in the Kaplan–Meier survival curves. (**D**) 1864 up-regulated in Type 2 T helper cell with the differential genes. (**E**) A total of 2331 up-regulated differential genes in activated CD4 T cells. (**F**) A total of 2322 up-regulated differential genes in activated dendritic cells. (**G**) ENO1 in Type 2 T helper by GSVA. (**H**) ENO1 in activated CD4 T cell by GSVA. (**I**) ENO1 in activated dendritic cell by GSVA. **** *p* < 0.0001.

Subsequently, we investigated the expression, survival, pathway enrichment, and activity of these five prognostic genes. Firstly, ENO1 expression was elevated in the tumor group (Figure 7A) and significantly higher in aging T cells compared to the young group (Figure 7B). Furthermore, high ENO1 expression in the Kaplan–Meier survival curves predicted a poorer prognosis (Figure 7C). ENO1 has been found to be overexpressed in a variety of cancers and is associated with tumor cell proliferation and metastasis [26,27]. Moreover, elevated STMN1 expression in both the tumor and senescent groups (Figure 7A,B) indicates worse outcomes in the Kaplan–Meier survival curves compared to the low expression group, suggesting a poor prognosis (Figure 7C). STMN1 is involved in tumorigenesis and tumor progression, with abnormal overexpression linked to aggressive tumor growth and poor prognosis [28]. Additionally, a similar trend was observed for PRDX1, RANBP1, and RAN genes.

In addition, there are three immune cells with significant differences based on high and low TERGs: activated CD4 T cells, activated dendritic cells, and Type 2 T helper cells. Specifically, 1864 upregulated genes were identified in Type 2 T helper cells (Figure 7D), 2331 upregulated differential genes in activated CD4 T cells (Figure 7E), and 2322 upregulated differential genes in activated dendritic cells (Figure 7F). Notably, elevated ENO1 expression in the tumor and aging groups significantly increased CD4-positive, α-β regulatory T cell differentiation within Type 2 T helper cells, enhancing PD-L1 expression and the PD-1 checkpoint pathway activity (Figure 7G). In contrast, in activated CD4 T cells, immune response pathways—including the T cell receptor complex, signaling pathway, differentiation of Th17, Th1, and Th2 cells, and MHC II peptide assembly—were significantly upregulated (Figure 7H). Importantly, MHC II molecule expression is crucial for initiating tumor immune responses by activating CD4+ T cells to target tumor cells. Meanwhile, the activity of pathways related to T cell activation and differentiation, including antigen processing and presentation in activated dendritic cells and T cell activation through the binding of T cell receptors to MHC molecules on antigen-presenting cells, was significantly higher in the high group compared to the low group (Figure 7I).

In addition, it is well established that Src, Lck, ZAP70, and LAT are direct interactors and potential components involved in T cell activation. It is observed that the expression levels of Lck, SRC, and LAT are significantly higher in the high group (Figure S2A–C). Lck, a key member of the Src tyrosine kinase family, primarily plays a role in the signal transduction of T cell receptors (TCRs) [29]. LAT functions as an adapter protein that integrates multiple signaling pathways and regulates T cell function through interactions with other signaling molecules [29,30,31]. In Figure S2D, there is no significant difference between the high and low groups; however, a trend towards higher values is observed. This suggests that the high group of TERGs is highly depleted, yet still enriched in most pathways associated with T cell activation. ZAP70 serves as a critical downstream signaling molecule in T cell activation; its deficiency can result in severe immunodeficiencies. These results indicate that our senescence-induced exhaustion T cells retain some effector function and may be reactivated by blocking the PD-L1 pathway [32].

### 2.8. Molecular Docking of Prognostic Genes with Targeted Drugs

**Figure 8:**
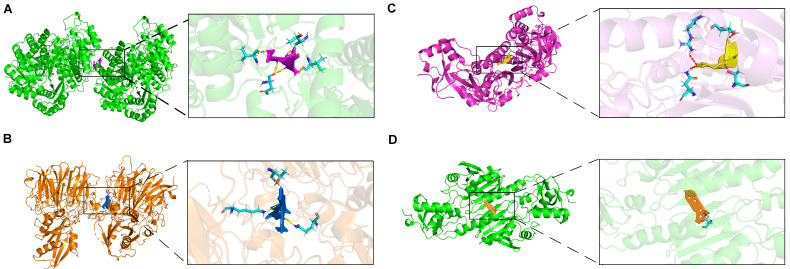
Molecular docking of targeted drugs. (**A**) The docking results of ENO1 with Gentamicin. (**B**) The docking results of RAN with Ivermectin. (**C**) The docking results of PRDX1 with Osteotriol. (**D**) The docking results of PRDX1 with Astragaloside C.

In this study, we investigated the role of prognostic genes in drug toxicology using the Comparative Toxicogenomics Database (CTD) and AutoDock molecular docking to identify potential drug targets among five prognostic genes. Our results indicate that ENO1 interacts with gentamicin, exhibiting a binding energy of −22.25 (kcal/mol), suggesting a strong affinity. This interaction may lead to the downregulation of gentamicin expression (Figure 8A). Molecular docking analysis revealed that no small molecule drugs targeting STMN1 and RANBP1 were identified. This limitation is attributed to the constraints of the protein structures in the CTD database. Future studies could utilize large models in deep learning drug screening to search for suitable drugs within extensive compound libraries (Figure S3). The effective docking energy of ivermectin with RAN was −15.64 (kcal/mol), suggesting its potential to reduce mRNA expression levels (Figure 8B).

Interestingly, the search for small molecule compounds that reduce PRDX1 expression identified osteotriol and Astragaloside C as promising candidates. Notably, osteotriol demonstrated a docking binding energy of −13.52 kcal/mol (Figure 8C). Astragaloside C effectively reduced PRDX1 expression, with a binding energy of −13.70 kcal/mol (Figure 8D). Also referred to as oleanolic acid, Astragaloside C is derived from Astragalus, a traditional Chinese medicine frequently utilized in the clinical management of hepatocellular carcinoma. Additionally, Astragalus polysaccharide essence (APS), an extract of Astragalus, has demonstrated anti-tumor effects in HCC. Research indicates that APS mediates its anti-tumor activity by modulating various signaling pathways, promoting apoptosis in tumor cells and enhancing immune response [33]. The combination of Astragalus polysaccharide essence (APS) with various chemotherapeutic agents can reduce drug side effects while enhancing anti-tumor efficacy. Several studies have demonstrated that APS possesses significant anti-inflammatory properties, inhibiting the expression and secretion of inflammatory cytokines such as TNF-α, IL-6, and MCP-1 [34].

## 3. Discussion

Hepatocellular carcinoma (HCC) is the fifth most common malignant tumor and a leading cause of cancer-related mortality worldwide [1]. Despite the diversity of treatment modalities for hepatocellular carcinoma (HCC), various options yield differing outcomes in patients with advanced disease.

A significant innovation in our study is the application of single-sample gene set enrichment analysis (ssGSEA) to cell clustering [35], enabling the classification of T cells into young and senescent subpopulations. This approach allowed us to analyze the complex components of the tumor microenvironment associated with senescence-induced exhausted T cells and to elucidate the relationship between senescent T cells and exhausted T cells.

Inflammatory aging refers to a sterile, low-grade chronic inflammation that gradually intensifies with advancing age. This phenomenon is linked to a wide range of age-related diseases, including cancer and neurodegenerative disorders [36,37,38,39]. In Results 5 and 7, pathways associated with Th17, Th1, and Th2 differentiation were highly enriched. Helper T cells, particularly Th17 cells, play a significant role in the pathogenesis of various inflammatory and autoimmune diseases [40]. More Treg cells were seen in the immune infiltration results of the high TERG group. Both helper T cell type 17 (Th17) and Treg cells are derived from naive CD4 T cells, and only undergo initial differentiation when stimulated by tumor growth factor (TGF) [41]. However, terminal differentiation leads to opposing cellular functions: Th17 cells promote autoimmunity and inflammation, while Treg cells inhibit immune responses and maintain immune homeostasis [41]. In the context of senescence-induced T-cell exhaustion, we hypothesize that, as senescence progresses within the organism, the activation of the pro-inflammatory factor Th17 contributes to inflammation. Concurrently, regulatory T cells (Treg) begin to suppress the immune response, leading to a diminished capacity for immune surveillance to eliminate senescent cells. This, in turn, promotes the development of inflammation [42]. In contrast, the balance between inflammation and suppression in the tumor immune microenvironment is regulated by many factors, including T cell receptors, co-stimulatory receptors, and cytokine-triggered signaling pathways [43]. This is consistent with the senescence-induced T-cell exhaustion pathway. We hypothesize that senescence-induced T-cell exhaustion can be delayed by regulating the balance between Th17 and Treg.

T-cell exhaustion refers to a state of T-cell dysfunction that occurs in many chronic infections and cancers [44]. Tregs are a subset of CD4+ T cells with immunosuppressive functions, crucial for maintaining self-tolerance and immune homeostasis [45]. In tumor immunity, Tregs impair immune surveillance of cancer in healthy individuals and suppress anti-tumor immune responses in the host, contributing to tumor progression across various cancer types [46]. Animal studies have demonstrated that the global depletion of Tregs enhances anti-tumor immunity and facilitates tumor rejection, with effective tumor immunity being induced in vivo through the depletion of CD25+CD4+ T cells [47]. In this study, we found that Tregs were increased in the tumor microenvironment, accompanied by high expression of PD-1, suggesting that exhausted T cells due to senescence contribute to an immunosuppressive environment. Moreover, PD-1 blockade of expanded PD-1 regulatory T cells promotes cancer progression [48]. Conversely, anti-PD-1 checkpoint therapy promotes the function and survival of regulatory T cells [49]. As a result, PD-1 immune blockade can reactivate cancer-killing T cells from their exhausted state. Antibodies that antagonize PD-1 are employed to restore T cell function and sustain their anti-tumor activity [45,50].

Previous studies have shown that PD-L1 expression inhibits cytotoxicity mediated by PD-1-expressing T cells. Additionally, elevated PD-L1 levels in senescent cells may enable these cells to evade immune surveillance, leading to their accumulation with age [51]. Thus, PD-L1 expression in senescent cells is essential for their escape from T cell-mediated immunity. This indicates that selectively inhibiting the accumulation of PD-L1-positive senescent cells may represent a more effective anti-aging strategy compared to conventional senescence therapies. Furthermore, anti-PD-L1 could serve as an ideal therapeutic target for both cancer treatment and anti-aging interventions, addressing the disease while preventing senescence. Over the past decade, research has increasingly supported the notion that blocking co-suppressor receptors’ binding capabilities can reverse T-cell exhaustion and thereby reactivate anti-tumor activity [52]. However, in recent years, perspectives on immune checkpoints have evolved significantly, with researchers positing that T-cell exhaustion is accompanied by profound changes in their epigenetic status, which are not easily reversible [53,54,55]. Thus, checkpoint blockade may primarily act to prevent the emergence of a depleted phenotype rather than reverse T-cell exhaustion, operating early within the T-cell terminal differentiation pathway [9]. Abrine, an immunosuppressant, can enhance the efficacy of anti-PD-1 antibody immunotherapy in hepatocellular carcinoma by effectively inhibiting immune escape [56].

In this study, two small molecule drugs showed promising results. Osteotriol targets the PRDX1 gene protein, exerting antitumor effects by modulating apoptosis and proliferation-related signaling pathways. Astragaloside C enhances immune cell proliferation and suppresses inflammation, leading to tumor cell apoptosis [57]. These findings present new strategies for cancer treatment and warrant further clinical investigation. In summary, repurposing existing “old drugs” can expedite the development of new treatments while simultaneously reducing research and development costs.

However, this study still has some limitations. Firstly, the sample size of patients with hepatocellular carcinoma was not sufficient. For this reason, more samples should be included to enrich the study when available. Secondly, the findings lack validation from multiple perspectives such as cells, tissues and animals. Moreover, the tumor microenvironment of HCC consists of a complex array of components, and multiple factors can affect the immune environment. This reflects the complexity of human immunity, and exploring the phenotypes that regulate tumor immunity is critical for modulating T cell responses. In the future, the emergence of new biomarkers and therapeutic approaches, the discovery of more potential drug targets, drug repurposing, drug combinations, and the elucidation of the mechanisms of the tumor immune microenvironment will be crucial for the development of immunotherapy, which will pave the way for better efficacy and effective treatment.

## 4. Materials and Methods

### 4.1. Data Collection

The scRNA-seq datasets of HCC were downloaded from the GEO database (https://www.ncbi.nlm.nih.gov/geo/, accessed on 5 April 2023), including GSE149614, GSE162616, GSE166635, and GSE175783. The GSE149614 dataset had data from 21 patients, including 10 patients with primary hepatocellular carcinoma tumor (PT), 2 patients with portal vein cancer thrombosis (PVTT), 1 patient with metastatic lymph node (MLN), and 8 patients with normal liver tissue (NLT). The GSE162616 dataset contains data from 13 patients, 2 patients from GSE166635, and 4 patients from GSE175783. For the TCGA-LIHC cohort, we downloaded transcriptomic data and clinical information for normal samples (*n* = 50) and HCC samples *(n* = 374) from the TCGA database (https://portal.gdc.cancer.gov/, accessed on 18 July 2023). The TCGA cohort was primarily used to analyze cell type-specific abundance, train and test sets, and to create prognostic exhaustion models.

### 4.2. Integration of Single-Cell Datasets, Standardization Process, and Cell Population Annotation

The single-cell dataset was analyzed using R version 4.3.2 (R version 4.3.2) with Seurat (v4.4.0). Firstly, the four scRNA-seq datasets were integrated and batch corrected by the Integrate Data function. The Percentage of mitochondria and rRNA was then calculated by the Percentage Feature Set function, with each cell expressing > 500 less than 5000 genes and ≥10% mitochondrial percentage content, and non-standard data were subsequently excluded. After filtration, the raw data contained a total of 28,458 genes and 229,458 cells. Next, they were processed through Seurat’s workflow. Using SingleR (V2.0.0) as well as the Cell Marker database (http://biocc.hrbmu.edu.cn/CellMarker/, accessed on 5 May 2023) and the PanglaoDB database (https://panglaodb.se/, accessed on 8 May 2023) Cell type annotation was performed.

### 4.3. The ssGSEA Algorithm Categorizes T Cells into Aging and Young T Cells

Firstly, the gene set for human senescence was downloaded from the database of Human Aging Genomic Resources (https://genomics.senescence.info/, accessed on 1 June 2023) and used to calculate the senescence index of T cells. Here we used single-sample Gene Set Enrichment Analysis (ssGSEA) for the functional enrichment analysis of different cells. The algorithm calculates the cumulative fraction of a specific functional gene in each cell by inputting a designated gene set, thereby indicating the degree of enrichment and correlation of each cell with that specific gene.

### 4.4. Comparison of Different Properties of Aging and Young T-Cell Subsets

AUCell, short for AUC-based Cells, is a tool for analyzing single-cell transcriptome data. It computes the cumulative distribution of gene expression levels and quantifies each gene’s expression pattern using the Area Under the Curve (AUC). By calculating AUC values for genes in individual cells, AUCell assesses the activity of relevant gene sets, aiding in the understanding of intracellular heterogeneity and functional status. In this study, we input a series of gene sets representing functional modules related to exhaustion, inflammation, and stress, and calculated the cumulative scores of the genes within these gene sets for each cell. In our study, we defined the three aforementioned scores based on a signature gene list that includes various genes associated with their corresponding pathways (see Table S2).

### 4.5. Screening of Gene Sets Highly Relevant to T-Cell Senescence

Pseudotime trajectories of T cell senescence were constructed using Monocle, which employs machine learning algorithms to arrange cells along trajectories with branching points based on specific input gene sets. This approach yields branches that represent distinct states within the cell population. The top 50 genes in these trajectories were identified using the FindAllMarkers function, designating them as T-cell exhaustion-related genes (TERGs).

### 4.6. Cibersort Calculates the Abundance of Cell Types in a Sample

CIBERSORT is a bioinformatics algorithm designed to calculate cell abundance by utilizing the expression matrix data of marker genes specific to particular cell types. It determines the relative proportion of these cell types within a given sample [58]. In this study, we used the marker genes of the top 50 T-cell subpopulations as inputs, aiming to compare the differential profiles of different cell types between normal and tumor tissues, and to train the model to predict the abundance of aging and young T-cell types in a sample. Then, differential genes for aging T cells were further calculated, defined as differential genes for T-cell exhaustion (DEGs). In addition, the results of cell abundance differences and correlation analysis were visualized by the “ggplot2” package.

### 4.7. Modeling Prognostic Exhaustion

We used an elastic network to construct a prognostic exhaustion model. The loss function of the Elastic Network Regression combines the regularization methods of Lasso Regression and Ridge Regression. The size of the penalty term is controlled by two parameters λ and ρ. In this study, we used caret and glmnet in R to select the optimal ρ and λ to identify reliable exhaustion signature genes. Meanwhile, we tested the performance of the model by subject work characteristic curves (ROC) and column line plots, and survival analysis to verify the validity of the prognostic exhaustion model, and thus assessed the accuracy and reliability of the model.

### 4.8. Functional Exploration of Prognostic Genes

To further investigate the function of prognostic genes in the model and try to elucidate the causes of poor prognosis, we grouped high and low according to the median number of TERGs. Then we analyzed the variability of the enriched pathways between the groups and analyzed the correlation of the pathway activities by Gene set variation analysis (GSVA).

Additionally, GECIP is an algorithm used to study the enrichment of pathways and the connections between related genes/proteins in gene expression data. GECIP utilizes metrics such as the connectivity of each gene, the frequency of genes within pathways, shared frequency, and overall connectivity. The final output is a calculation of the predicted crosstalk degree (CTdegree) between pathways, indicating potential interactions that may exist among enriched pathways [59].

### 4.9. Screening of Targeted Drugs

In AutoDock molecular docking, the criteria for the delineation of molecular compounds have two main aspects. On the one hand, the magnitude of the binding energy value serves as a crucial indicator for assessing the quality of molecular docking. It represents the strength of intermolecular interactions and is commonly utilized to evaluate the degree of molecular binding to target proteins, thereby predicting the ligand’s affinity for the target. A lower binding energy value indicates a tighter binding between the molecule and the target protein, with more negative binding energy values typically associated with better docking efficacy. On the other hand, from a molecular structural perspective, the deeper the penetration of molecular drug chimeras into the pockets of protein structural domains, the better the outcomes of drug-target docking interactions. We used Autodock (Linux, v5.15.0) for molecular docking to target small molecule compounds that interact with prognostic genes. First, we downloaded information on small molecule drugs from the CTD database (http://ctdbase.org/, accessed on 15 November 2023), and then the structures of the corresponding small molecule compounds were downloaded from the PubChem database (https://pubchem.ncbi.nlm.nih.gov/, accessed on 16 November 2023). Next, we searched and downloaded the structures of biomolecules translated by prognostic gene transcription from the Uniport database (https://www.uniprot.org, accessed on 17 November 2023). Finally, the docking of biomacromolecules and small molecule drugs was accomplished in Linux. As a result, the small molecules with the lowest binding energy for binding to biomolecules were screened. In addition, the results were visualized by Pymol (V2.6, open source).

### 4.10. Statistical Analysis

Statistical analysis was performed using R version 4.3.2. For between groups, non-parametric tests were used. For the significance analysis of the probability of survival between samples, the log rank test was used and a statistically significant difference was considered at *p* < 0.05. In addition, for correlation analysis, Spearman rank correlation analysis was used.

## 5. Conclusions

This research provides a comprehensive analysis of the tumor immune microenvironment in hepatocellular carcinoma (HCC), focusing on the interplay between exhausted T cells and senescent T cells. By integrating and analyzing single-cell RNA sequencing (sRNA-seq) data, the study delineates the distinct characteristics of senescent and young T cells, revealing an immunosuppressive environment in HCC patients. The findings underscore the roles of inflammation-induced senescence, T-cell exhaustion, and immune checkpoints (notably PD-1) in HCC progression. While targeting PD-L1-positive senescent cells shows potential for both anticancer and anti-aging therapies, significant challenges remain in preventing T-cell exhaustion and fully understanding the complex tumor immune microenvironment.

## Data Availability

The datasets analyzed in this study are available in GEO and TCGA repositories, including GSE162616, GSE166635, GSE175783, GSE149614 and TCGA-LIHC. The code used in this study is available upon request by contacting the corresponding author.

## Supplementary Materials

The following supporting information can be downloaded at: https://www.mdpi.com/article/10.3390/ijms252011232/s1.

## List of Abbreviations

HCC	Hepatocellular Carcinoma
LIHC	Liver hepatocellular carcinoma
TIME	Immune microenvironment
scRNAseq	Single-cell RNA sequencing
PT	primary hepatocellular carcinoma tumor
PVTT	portal vein cancer thrombosis
MLN	metastatic lymph node
NLT	normal liver tissue
TREGs	T-cell exhaustion-related genes
GEO	Gene Expression Omnibus Database
TCGA	The Cancer Genome Atlas
GSEA	Gene Set Enrichment Analysis
ROC	Receiver operating characteristic curve
ssGSEA	Single sample Gene Set Enrichment Analysis
GSVA	Gene set variation analysis
CTD	Comparative Toxicogenomics Database
Treg Cell	Regulatory T Cell
Th17	T helper type 17 cell
