# Supplementary figures and images for: Targeted Drug Screening Leveraging Senescence-Induced T-Cell Exhaustion Signatures in Hepatocellular Carcinoma
Source: Int J Mol Sci. 2024 Oct 18;25(20):11232. doi: 10.3390/ijms252011232 (PMC11508728; doi:10.3390/ijms252011232)

**A**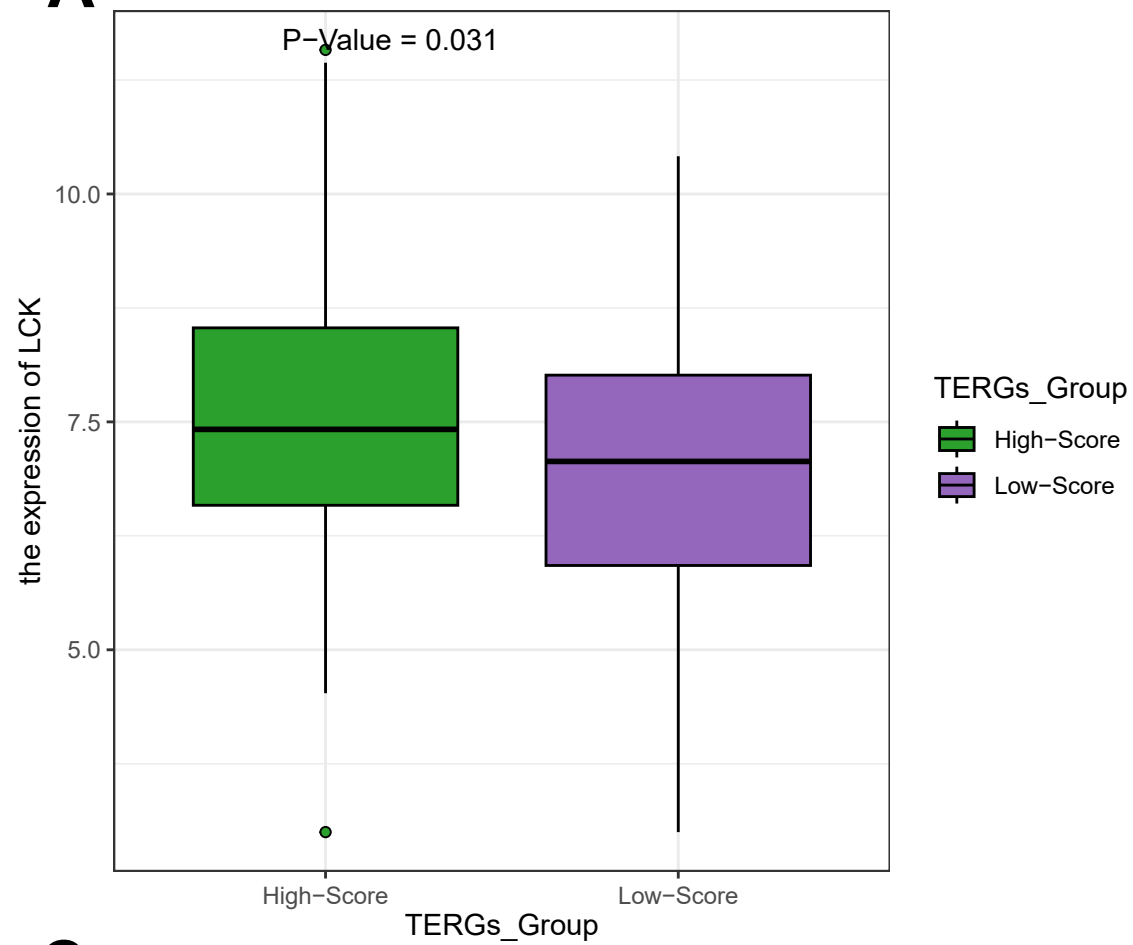**B**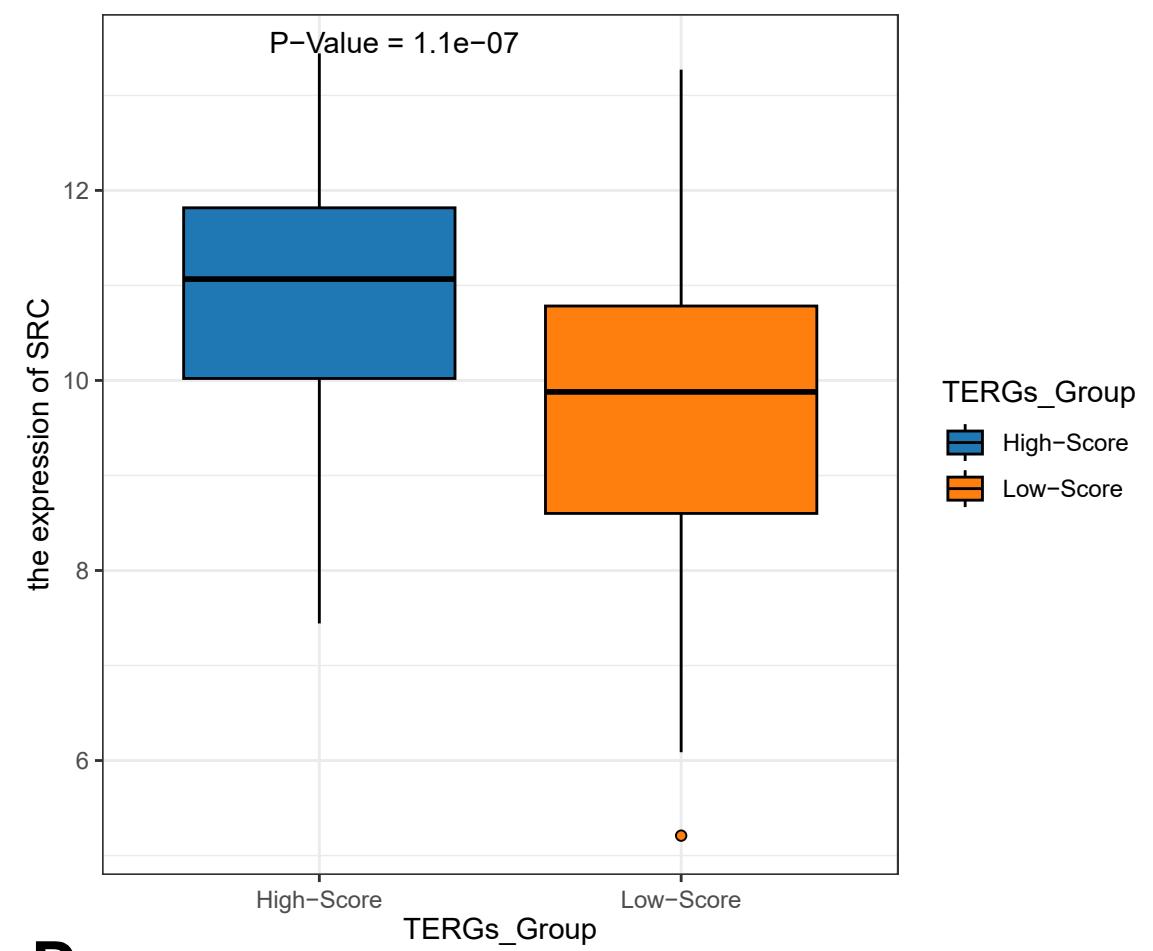**C**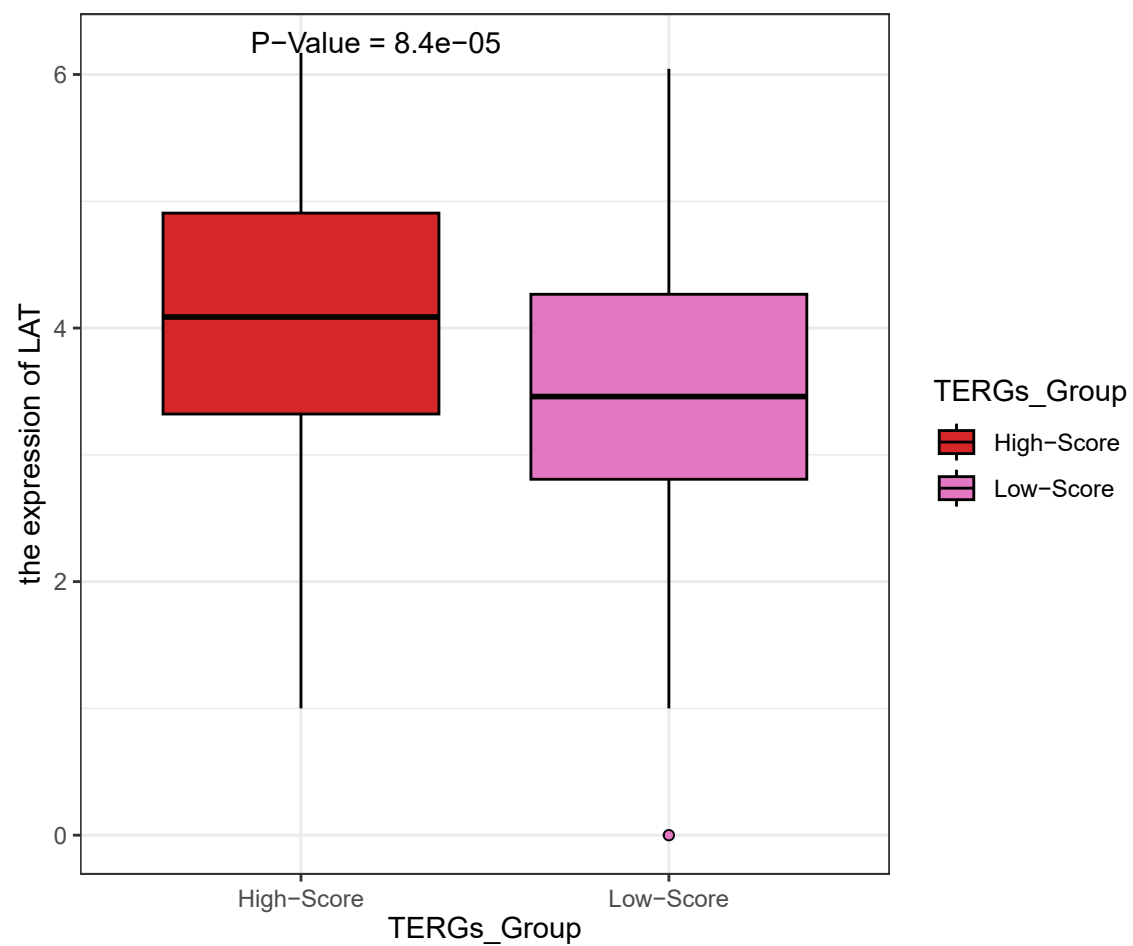**D**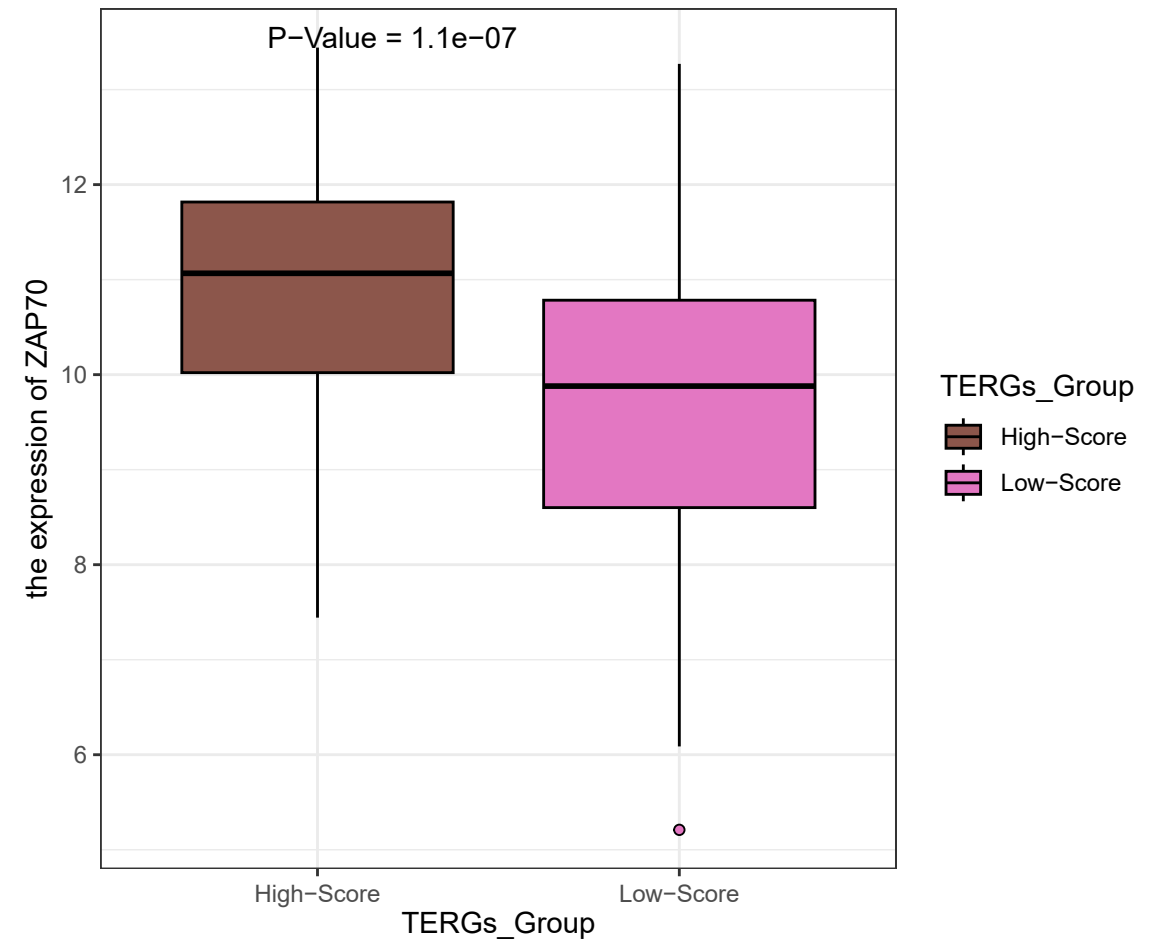

Supplement: Supplementary file 1 [file ijms-25-11232-s001.zip › Supplementary File/Figure S2.pdf]

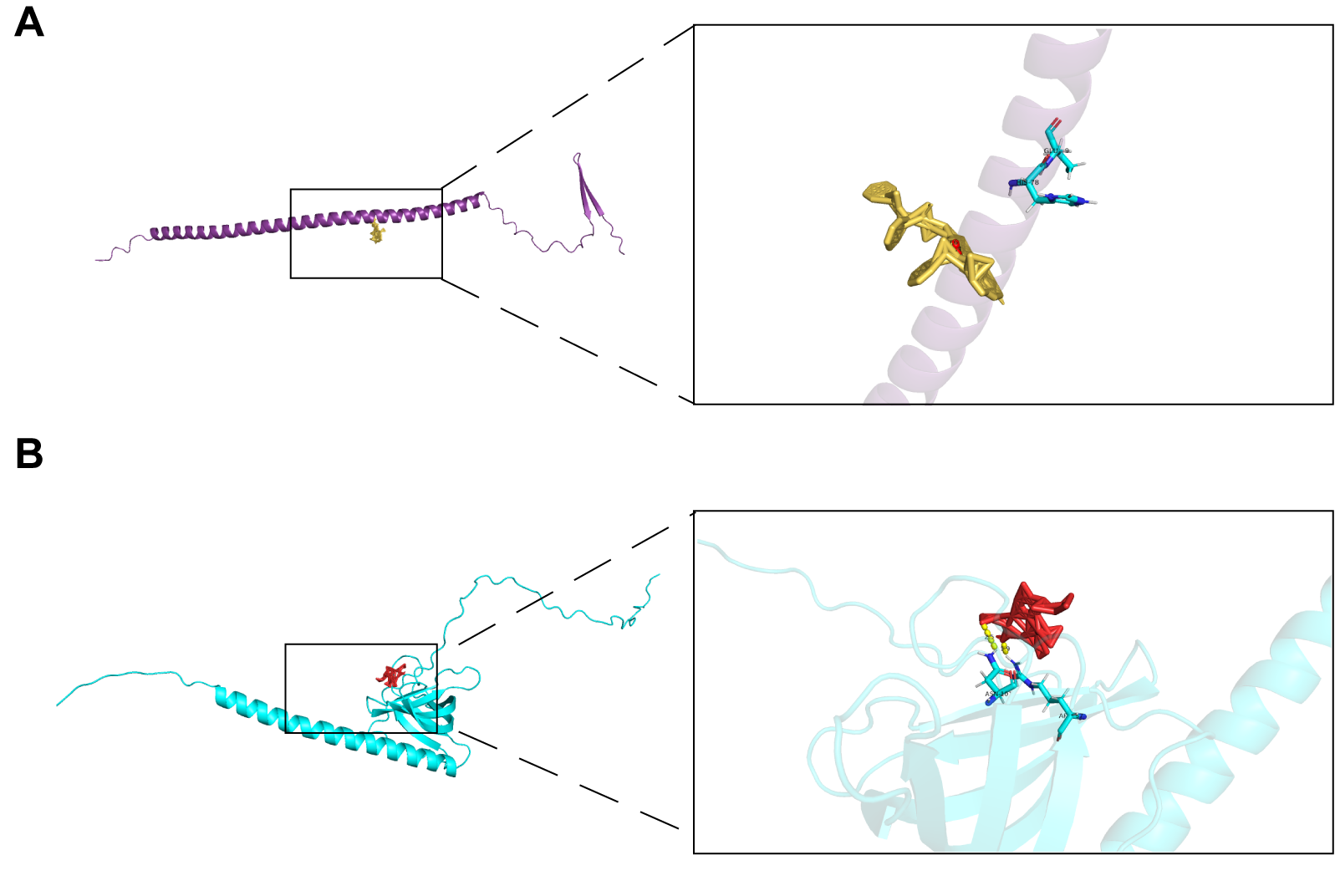

Supplement: Supplementary file 1 [file ijms-25-11232-s001.zip › Supplementary File/Figure S3.tif]
